# Avaliação do perfil imunológico de adultos mais velhos segundo as regiões brasileiras: resultados do ELSI-Brasil

**DOI:** 10.1590/0102-311XPT172124

**Published:** 2026-05-29

**Authors:** Maria Luiza Lima-Silva, Juliana Vaz de Melo Mambrini, Karen Cecília de Lima Torres, Olindo Assis Martins-Filho, Andréa Teixeira-Carvalho, Maria Fernanda Lima-Costa, Sérgio Viana Peixoto

**Affiliations:** 1 Instituto René Rachou, Fundação Oswaldo Cruz, Belo Horizonte, Brasil.; 2 Núcleo de Estudos em Saúde Pública e Envelhecimento, Fundação Oswaldo Cruz/Universidade Federal de Minas Gerais, Belo Horizonte, Brasil.; 3 Grupo Integrado de Pesquisa em Biomarcadores, Fundação Oswaldo Cruz, Belo Horizonte, Brasil.; 4 Universidade Edson Antônio Velano, Belo Horizonte, Brasil.; 5 Escola de Enfermagem, Universidade Federal de Minas Gerais, Belo Horizonte, Brasil.

**Keywords:** Imunossenescência, Biomarcadores, Envelhecimento, Saúde do Idoso, Immunosenescence, Biomarkers, Aging, Health of the Elderly, Inmunosenescencia, Biomarcadores, Envejecimiento, Salud de Anciano

## Abstract

A imunossenescência refere-se às alterações do sistema imunológico com o envelhecimento, incluindo o aumento dos biomarcadores inflamatórios, fenômeno conhecido como *inflammaging*, que está relacionado ao surgimento de doenças associadas ao envelhecimento. O objetivo deste estudo foi descrever a distribuição de biomarcadores imunológicos nas cinco regiões brasileiras e estimar as probabilidades preditas de suas concentrações nos tercis da distribuição, segundo as regiões geográficas. Amostras sorológicas de 2.111 participantes (≥ 50 anos) coletadas na linha de base (2015/2016) do ELSI-Brasil foram analisadas. As regiões geográficas (Norte, Nordeste, Centro-oeste, Sudeste e Sul) foram a exposição de interesse do estudo, e os potenciais fatores de confusão incluíram idade, sexo, nível educacional, área de residência, consumo de álcool e tabaco, atividade física e autodeclaração de diagnósticos médicos de hipertensão, diabetes, asma, artrite e câncer. As probabilidades preditas das concentrações dos biomarcadores em cada tercil foram estimadas a partir de modelos de regressão logística multinomial, ajustados pelos confundidores. Notou-se uma maior probabilidade de as regiões Norte e Nordeste apresentarem níveis séricos mais altos, enquanto a Região Centro-oeste níveis mais baixos, sugerindo diferenças na imunobiografia que podem ser resultado de exposições ambientais, socioeconômicas e infecciosas distintas ao longo da vida. No entanto, as razões para essas diferenças ainda não foram elucidadas e precisam ser investigadas em estudos posteriores.

## Abstract (en)

Immunosenescence refers to the changes in the immune system with aging, including the increase in inflammatory biomarkers, a phenomenon known as Inflammaging, which is related to the emergence of diseases associated with aging. This study aimed to describe the distribution of immunological biomarkers in the five Brazilian regions and to estimate the predicted probabilities of their concentrations in the tertiles of distribution, according to geographic regions. Serological samples from 2,111 participants (≥ 50 years) collected at the baseline (2015/2016) of ELSI-Brazil were analyzed. The geographic regions (North, Northeast, Central-West, Southeast, and South) were the exposure of interest for the study, and potential confounding factors included age, sex, educational level, area of residence, alcohol and tobacco consumption, physical activity, and self-reported medical diagnoses of hypertension, diabetes, asthma, arthritis, and cancer. The predicted probabilities of biomarker concentrations in each tertile were estimated using multinomial logistic regression models, adjusted for confounders. Notably, the North and Northeast regions had higher serum levels, while the Central-West Region had lower levels, suggesting differences in immunobiography, which may be the result of different environmental, socioeconomic, and infectious exposures throughout life. However, the reasons for these differences have not yet been elucidated and need to be investigated in further studies.

## Abstract (es)

La inmunosenescencia se refiere a las alteraciones en el sistema inmunológico con el envejecimiento, incluido el aumento de los biomarcadores inflamatorios, un fenómeno conocido como *inflammaging*, que está relacionado con la aparición de enfermedades relacionadas al envejecimiento. El objetivo de este estudio fue describir la distribución de biomarcadores inmunológicos en las cinco regiones brasileñas y estimar las probabilidades previstas de sus concentraciones en los terciles de la distribución, según las regiones geográficas. Se analizaron muestras serológicas de 2.111 participantes (≥ 50 años) recolectadas al inicio (2015/2016) del ELSI-Brasil. Las regiones geográficas (Norte, Nordeste, Centro-oeste, Sudeste y Sur) fueron la exposición de interés del estudio, y los posibles factores de confusión incluyeron edad, sexo, nivel educativo, área de residencia, consumo de alcohol y tabaco, actividad física y diagnósticos médicos autoinformados de hipertensión, diabetes, asma, artritis y cáncer. Las probabilidades previstas de concentraciones de biomarcadores en cada tercil se estimaron con base en modelos de regresión logística multinomial, ajustados por factores de confusión. Se observó que las regiones Norte y Nordeste tenían mayor probabilidad de presentar niveles séricos más elevados, mientras que la Región Centro-oeste mostró niveles más bajos, lo que sugiere diferencias en la inmunobiografía, que pueden ser el resultado de distintas exposiciones ambientales, socioeconómicas e infecciosas a lo largo de la vida. Sin embargo, las razones de estas diferencias aún no se han dilucidado y es necesario investigarlas en estudios posteriores.

## Introdução

O envelhecimento do sistema imune, também chamado de imunossenescência, é marcado por um remodelamento de funções biológicas, como a involução tímica, a maior prevalência de células de memória e pior resposta a antígenos, além do aumento de biomarcadores inflamatórios ^1^ ^,^ ^2^ ^,^ ^3^. Esse último pode ser englobado ao *inflammaging*, processo de inflamação crônica de baixo grau que tem um papel importante durante o envelhecimento e pode contribuir para o desenvolvimento de doenças crônicas, como artrite e diabetes ^4^ ^,^ ^5^ ^,^ ^6^.

A maior parte dos estudos que avaliaram de forma representativa as características do sistema imunológico de adultos mais velhos (50 anos ou mais) foi realizada em países de alta renda, tais como a Inglaterra (*English Longitudinal Study of Ageing* − ELSA) e os Estados Unidos (*Health and Retirement Study*) ^7^ ^,^ ^8^ ^,^ ^9^. Pesquisas com essa temática e representatividade populacional realizadas em países de baixa e média renda são escassas. No Brasil, até onde sabemos, o único estudo que avaliou o perfil imunológico de adultos mais velhos, nacionalmente representativo, foi conduzido na população do *Estudo Longitudinal de Saúde dos Idosos Brasileiros* (ELSI-Brasil) ^10^. Este trabalho é harmonizado com aqueles supracitados, entretanto, não foram consideradas as diferenças regionais.

Quando comparada a de outros países, a população brasileira apresenta uma série de peculiaridades, como a maior prevalência de doenças infecciosas tropicais ^11^ ^,^ ^12^ ^,^ ^13^ e as características genéticas multiétnicas ^14^ ^,^ ^15^ ^,^ ^16^, além do contexto de desigualdades sociais que impactam o sistema de saúde ^17^ ^,^ ^18^ ^,^ ^19^.

Ademais, o Brasil é um país com dimensões continentais e apresenta diferenças importantes entre suas cinco grandes regiões geográficas (Norte, Nordeste, Centro-oeste, Sudeste e Sul). A população residente nos estados das regiões Sul e Sudeste, em geral, tem melhores condições socioeconômicas em detrimento das outras, enquanto a Região Nordeste, comparada com as demais, tem menores níveis de escolaridade, de acordo com o último censo do Instituto Brasileiro de Geografia e Estatística (IBGE) ^20^ ^,^ ^21^. Somam-se a essas diferenças desigualdades das condições relacionadas à saúde, como piores indicadores de alimentação saudável e prática de atividade física no Norte e Nordeste ^22^, melhor avaliação da atenção primária em saúde (APS) na Região Sul ^23^ e declínio das médias das taxas de mortalidade municipais por doenças crônicas não transmissíveis no Sul, Sudeste e Centro-oeste, enquanto o Nordeste apresentou acréscimo nesse parâmetro (2010 a 2017) ^24^. Em relação aos fatores ambientais, a Região Sudeste apresentou a maior taxa de mortalidade atribuível à poluição do ar, entre 2010 e 2019, ao passo que a Região Norte ocupou a segunda posição ^25^. Por outro lado, a Região Nordeste apresentou a maior taxa de mortalidade por serviços inadequados de WASH (água, saneamento e higiene, do inglês: *water, sanitation and hygiene*), enquanto a Região Sul apresentou a menor taxa em 2020 ^26^. De modo geral, a Região Norte concentra indicadores de saúde relativamente mais distantes das demais regiões geográficas brasileiras.

Essas diferenças impactam a imunobiografia, perfil de resposta imune entendido como o reflexo de modelações e adaptações ocorridas ao longo da vida, e resultado dos estímulos aos quais os organismos foram expostos ^27^. Portanto, indivíduos expostos a distintos estressores ambientais apresentam diferenças em seu perfil de resposta imune ^28^. Sendo o Brasil um país de dimensões continentais e considerando as marcantes diferenças entre suas regiões, é relevante se conhecer o processo de *inflammaging*, na perspectiva da imunobiografia. Assim, compreender o perfil imunológico nas diferentes regiões brasileiras pode fornecer subsídios para propor intervenções mais assertivas e regionais no âmbito do Sistema Único de Saúde (SUS), visando melhorar a qualidade de vida dos adultos mais velhos. Hipotetiza-se que existem diferenças no perfil de concentração dos biomarcadores entre as regiões brasileiras, bem como em relação aos países desenvolvidos. Assim, o objetivo deste estudo foi descrever a distribuição de biomarcadores imunológicos nas cinco regiões brasileiras e estimar as probabilidades preditas das concentrações de cada biomarcador nos tercis da distribuição, segundo as regiões geográficas.

## Metodologia

### População do estudo

Esta pesquisa foi uma análise transversal que contou com a utilização de dados da linha de base do ELSI-Brasil, realizada em 2015-2016 em uma amostra nacionalmente representativa de indivíduos com 50 anos ou mais não institucionalizados. O desenho amostral foi baseado em estratos, considerando os municípios, setores censitários e domicílios. A amostra final da linha de base do ELSI-Brasil incluiu 9.412 indivíduos, residentes em 70 municípios das cinco regiões do país. A coleta de sangue foi realizada em uma subamostra representativa de 2.361 participantes. Mais detalhes sobre o desenho geral do ELSI-Brasil e da amostra para coleta sanguínea podem ser encontrados no trabalho de Lima-Costa et al. ^29^.

## Aspectos éticos

O ELSI-Brasil foi aprovado pelo Comitê de Ética em Pesquisa do Instituto René Rachou, Fundação Oswaldo Cruz, Minas Gerais (CAAE 34649814.3.0000.5091), e todos os entrevistados assinaram termos de consentimento livre e esclarecidos para participar do estudo.

### Variáveis do estudo

#### Desfechos

Os desfechos foram as concentrações dos biomarcadores séricos, avaliados por meio de detecção e quantificação simultâneas pelo método Luminex 27-plex de alto desempenho (Bio-Rad Laboratories, Hercules, Estado Unidos, número do lote: 64104297. https://www.bio-rad.com/). Os biomarcadores avaliados incluíram: citocinas (IFN-γ, IL-1β, IL-1Ra, IL-4, IL-5, IL-6, IL-9, IL-10, IL-12, IL-13, IL-15, IL-17 e TNF-α), quimiocinas (CXCL8, CXCL10, CCL2, CCL3, CCL4, CCL5 e CCL11) e fatores de crescimento (FGF, G-CSF, GM-CSF, IL-2, IL-7, PDGF e VEGF). O coeficiente de variação intra e entre ensaios é de 0-8% e 0,1-15,5%, respectivamente. Os limites de detecção variam entre 0,2 e 19,3pg/mL, de acordo com o fabricante. As amostras de soro de 2.111 participantes foram analisadas seguindo as instruções do fabricante, utilizando reagentes, padrões e placas. As dosagens não realizadas de 250 amostras estavam relacionadas à material insuficiente ou hemolisado. Durante o ensaio, foi adquirido um mínimo de 50 microesferas por biomarcador, no equipamento Luminex 200 (Bio-Rad), por meio da intensidade média de fluorescência para cada biomarcador. As concentrações de cada amostra foram determinadas com base em curvas padrão executadas para cada molécula, empregando uma análise de ajuste do quarto ou quinto parâmetro logístico. Amostras que apresentaram valores de concentração abaixo da extrapolação desses parâmetros logísticos receberam um valor de 0,0001pg/mL. Por outro lado, amostras com valores acima do limite superior de quantificação foram substituídas pelo maior nível de concentração sérica quantificada para o respectivo biomarcador. O desempenho das características, incluindo limite inferior ou superior de quantificação, coeficiente de variação e sensibilidade do ensaio, pode ser acessado no guia *Analyte do Bio-PlexPro Multiplex Immunoassays* (https://www.bio-rad.com/webroot/web/pdf/lsr/literature/Bulletin6335.pdf). Os resultados foram expressos em pg/mL para todos os biomarcadores testados.

#### Variáveis exploratórias

A exposição de interesse do estudo foram as regiões geográficas (Norte, Nordeste, Centro-oeste, Sudeste e Sul). Os potenciais fatores de confusão foram selecionados com base na literatura e sua relevância para os parâmetros imunológicos ^10^ ^,^ ^17^ ^,^ ^29^ ^,^ ^30^, visando controlar o efeito dos fatores que apresentam diferenças importantes entre as regiões. Esses fatores incluíram faixa etária (50-59 anos, 60-69 anos, 70-79 anos e 80 anos ou mais), sexo (masculino e feminino), escolaridade (menos de 4, 4 a 7, ou 8 anos de estudo ou mais) e área de residência (rural ou urbana). Como hábitos de vida, foram considerados o consumo de álcool (abstêmios, consumo moderado e consumo de risco), tabagismo (não fumante, ex-fumante e fumante atual), prática de atividade física (< 150 minutos por semana e ≥ 150 minutos por semana) e diagnósticos médicos autorrelatados de doenças crônicas, incluindo hipertensão, diabetes, asma, artrite e câncer.

O consumo de álcool foi avaliado conforme as diretrizes do Instituto Nacional sobre Abuso de Álcool e Alcoolismo ^31^, considerando a frequência e quantidade ingerida. Participantes que consumiam álcool menos de uma vez por semana ou não consumiam foram considerados abstêmios. O consumo moderado foi definido como até 7 doses semanais para mulheres e até 14 para homens, enquanto quantidades maiores foram classificadas como de alto risco. Além disso, mulheres que consumiam 4 ou mais doses por ocasião, e homens que consumiam 5 ou mais, foram classificados como bebedores pesados e de alto risco. O tabagismo atual foi avaliado pelo relato do hábito de fumar no momento da entrevista, independente da frequência. Foram considerados ex-fumantes, os indivíduos que responderam ter fumado no passado, mas não no momento da entrevista. Os demais foram classificados como nunca fumantes. A atividade física foi mensurada utilizando a versão curta do *Questionário Internacional de Atividade Física* (IPAQ, acrônimo em inglês), traduzido e validado para o Brasil. Considerou-se a frequência (dias por semana) e a duração (tempo por dia) da atividade física realizada por pelo menos 10 minutos contínuos na semana anterior à entrevista. Em relação à atividade física, os participantes foram classificados como aqueles que praticavam atividade física de forma suficiente (150 minutos ou mais, por semana) ou insuficiente (< 150 minutos por semana), de acordo com o nível regular preconizado ^32^.

### Análise estatística

A descrição das características dos participantes foi realizada para a população total e segundo as regiões geográficas com base na distribuição relativa. Para avaliar as diferenças das características sociodemográficas, comportamentos em saúde e doenças crônicas entre as regiões geográficas foi utilizado o teste qui-quadrado de Pearson com correção de Rao-Scott. A distribuição dos níveis de biomarcadores por regiões foi apresentada a partir da mediana e intervalos interquartílicos em pg/mL. Para avaliar a existência de diferenças entre as regiões foi utilizado o teste não-paramétrico de Kruskall-Wallis.

Foi utilizado o modelo de regressão logística multinomial para estimar os *odds ratio* (OR) e seus respectivos intervalos de 95% de confiança (IC95%). A distribuição dos biomarcadores foi categorizada em tercis, considerando o 1º tercil e a Região Norte como categorias de referência, com ajuste pelas variáveis exploratórias. Os resultados dessa análise foram incluídos na Tabela S1 (Material Suplementar; S1: https://cadernos.ensp.fiocruz.br/static//arquivo/suppl-e00172124_2029.pdf) e foram usados apenas para produzir estimativas de probabilidades, ajustadas pelos fatores de confusão, objetivo do presente artigo.

A partir dos modelos multinomiais ajustados, foram então calculadas as probabilidades preditas de os indivíduos estarem em cada tercil para cada biomarcador e região, permitindo-se conhecer o padrão de distribuição dos biomarcadores entre as regiões brasileiras. Os resultados foram apresentados em um *heatmap*.

As análises estatísticas foram realizadas no software Stata versão 14.0 (https://www.stata.com) e R versão 4.1.1 (http://www.r-project.org), com o uso das bibliotecas *ggplot2* ^33^, *RcolorBrewer* e *survey* ^34^ ^,^ ^35^. O efeito do desenho amostral complexo da pesquisa foi considerado para as subamostras sanguíneas em todos os testes estatísticos, e foi adotado o nível de 5% de significância.

## Resultados

Participaram do estudo 2.111 indivíduos de todas as regiões geográficas do país. Residiam na Região Norte 3,1% dos participantes, 24,5% no Nordeste, 7,7% no Centro-oeste, 46,1% no Sudeste e 18,6% na Região Sul.

A Tabela 1 descreve as variáveis incluídas no presente estudo, para a amostra total e por região de residência. Para a amostra analisada, houve predominância do sexo feminino (52,7%) e dos grupos etários mais jovens, além disso, 62,4% da população tinha menos de 4 anos de escolaridade. Com relação aos hábitos de vida, 83% eram abstêmias, 45,2% nunca fumaram e 34,2% não praticavam atividade física em níveis suficientes. As prevalências para doenças crônicas foram 25,5% para artrite, 4,8% para asma, 6% para câncer, 16,6% para diabetes e 52,9% para hipertensão. Diferenças significativas entre regiões foram observadas para as variáveis de comportamentos e relato de diagnóstico médico para câncer.

**Tabela 1:** Características sociodemográficas na amostra estudada por regiões brasileiras. *Estudo Longitudinal da Saúde dos Idosos Brasileiros* (ELSI-Brasil), 2015-2016.

Variáveis	Total	Norte	Nordeste	Centro-oeste	Sudeste	Sul	Valor de p
Sexo							
Feminino	52,7	49,2	52,9	51,7	52,3	54,3	0,908
Masculino	47,3	50,8	47,1	48,3	47,7	45,7	
Grupos etários (anos)							
50-59	46,1	38,7	44,4	48,2	45,9	49,1	0,978
60-69	30,5	32,4	30,5	31,3	30,8	29,3	
70-79	15,9	18,9	17,5	14,8	15,3	15,3	
≥ 80	7,5	10,0	7,6	5,7	8,0	6,3	
Escolaridade (anos)							
< 4	62,4	76,0	68,9	72,2	59,4	55,0	0,353
4-7	9,8	13,6	9,0	8,7	8,6	13,9	
8 ou mais	27,8	10,4	22,1	19,1	32,0	31,1	
Zona							
Rural	9,9	33,7	14,4	0,0	8,7	6,8	0,316
Urbana	90,1	66,4	85,6	1,0	91,3	93,2	
Consumo de álcool							
Abstêmio	83,0	89,3	83,6	84,5	80,4	87,0	0,048
Moderado	6,8	2,5	3,2	2,6	9,7	6,8	
Consumo de risco	10,2	8,3	13,3	12,9	9,9	6,3	
Tabagismo							
Nunca fumou	45,2	45,5	40,6	40,2	46,5	50,3	0,006
Ex-fumante	39,0	45,5	43,9	48,5	39,1	27,4	
Fumante atual	15,8	9,0	15,5	11,3	14,5	22,4	
Prática de atividade física							
Suficiente	65,8	55,5	53,5	78,1	70,4	67,3	0,034
Insuficiente	34,2	44,5	46,5	21,9	29,6	32,7	
Condições de saúde							
Artrite	25,5	32,9	23,0	26,5	25,2	27,8	0,652
Asma	4,8	4,6	3,6	6,0	5,6	4,0	0,495
Câncer	6,0	2,5	3,5	3,5	6,9	8,6	0,048
Diabetes	16,6	11,4	17,0	17,9	17,4	14,5	0,613
Hipertensão arterial	52,9	50,6	52,2	57,9	53,5	50,9	0,726

Nota: todas as estimativas consideraram o desenho amostral complexo e os pesos da pesquisa. Valor de p estimado pelo teste qui-quadrado com correção de Rao-Scott.

A distribuição da concentração das quimiocinas, das citocinas e dos fatores de crescimento nos participantes do ELSI-Brasil, segundo região geográfica, está apresentada na Tabela 2. Essa análise revelou que existem diferenças, entre as regiões brasileiras, para todos os biomarcadores solúveis, com exceção da citocina reguladora IL-4 e dos fatores de crescimento G-CSF e IL-2 (Tabela 2).

**Tabela 2:** Concentração de biomarcadores imunológicos na amostra estudada por regiões brasileiras. *Estudo Longitudinal da Saúde dos Idosos Brasileiros* (ELSI-Brasil), 2015-2016.

Biomarcadores	Total	Norte	Nordeste	Centro-oeste	Sudeste	Sul	Valor de p *
Quimiocinas							
CXCL8	22,01 (7,05-112,38)	19,87 (9,13-114,02)	22,01 (9,35-81,66)	6,90 (4,5-21,04)	26,05 (7,88-157,84)	24,07 (6,81-149,92)	0,001
CCL11	43,93 (25,99-65,44)	47,63 (24,32-71,12)	48,88 (30,39-78,48)	41,52 (24,78-61,19)	42,36 (24,44-60,12)	43,1 (25,38-59,88)	< 0,001
CCL3	2,52 (1,39-7,32)	4,48 (1,93-12,74)	3,21 (1,74-8,34)	1,62 (0,91-2,38)	2,49 (1,48-7,09)	1,84 (0,99-11,06)	< 0,001
CCL4	14,19 (9,61-23,33)	16,77 (11,01-28,22)	17,30 (11,68-28,49)	12,66 (8,49-17,52)	13,33 (9,61-22,18)	11,85 (8,82-23,33)	0,012
CCL2	19,33 (9,57-35,90)	21,87 (13,05-41,11)	27,10 (12,21-44,88)	18,31 (8,73-9,43)	16,33 (8,76-30,19)	19,69 (7,32-33,41)	0,001
CCL5	603,46 (402,24-1.024,63)	644,27 (409,62-1.183,82)	882,26 (552,76-2.715,25)	946,84 (480,59-2.188,68)	526,49 (346,78-817,57)	537,74 (396,57-813,00)	< 0,001
CXCL10	55,78 (32,9-91,01)	61,25 (43,94-94,65)	78,81 (46,44-142,49)	55,43 (33,68-118,69)	50,63 (32,54-74,54)	49,58 (23,91-79,47)	< 0,001
Citocinas inflamatórias							
IL-1β	0,31 (0,20-0,58)	0,28 (0,19-0,53)	0,36 (0,24-0,69)	0,26 (0,14-0,41)	0,28 (0,18-0,48)	0,42 (0,24-1,04)	0,004
IL-6	0,25 (0,14-0,37)	0,29 (0,23-0,40)	0,30 (0,14-0,42)	0,18 (0,001-0,28)	0,24 (0,14-0,33)	0,30 (0,17-0,39)	< 0,001
TNF-α	3,21 (1,69-4,99)	3,35 (1,67-4,84)	3,88 (2,71-5,44)	2,76 (1,00-4,25)	2,55 (1,30-4,31)	3,83 (2,82-5,62)	< 0,001
IL-12	0,39 (0,25-0,52)	0,41 (0,37-0,53)	0,36 (0,12-0,52)	0,34 (0,001-0,51)	0,39 (0,26-0,52)	0,41 (0,34-0,56)	< 0,001
IFN-γ	0,66 (0,36-1,06)	0,69 (0,33-1,13)	0,77 (0,44-1,28)	0,41 (0,001-0,79)	0,64 (0,41-1,02)	0,73 (0,33-1,06)	0,001
IL-15	13,82 (0,001-27,52)	19,89 (4,48-33,9)	24,42 (11,75-39,29)	15,40 (0,001-27,26)	9,33 (0,001-21,85)	11,75 (0,001-27,26)	< 0,001
IL-17	3,02 (20,01-4,69)	3,14 (2,45-4,96)	3,28 (2,11-5,13)	2,49 (1,65-3,55)	2,73 (1,86-4,25)	3,66 (2,11-5,59)	0,011
Citocinas reguladoras							
IL-1Ra	121,02 (68,48-215,24)	163,29 (88,14-358,93)	121,02 (70,59-195,28)	110,35 (66,67-177,09)	126,75 (76,05-221,59)	114,02 (57,03-284,68)	0,009
IL-4	0,97 (0,64-1,37)	1,01 (0,75-1,34)	1,06 (0,67-1,51)	0,95 (0,61-1,16)	0,94 (0,62-1,28)	1,10 (0,69-1,43)	0,354
IL-5	1,46 (0,36-2,23)	1,68 (1,01-2,46)	1,89 (0,28-2,57)	0,99 (0,001-1,82)	1,19 (0,09-2,06)	1,73 (1,13-2,42)	< 0,001
IL-9	4,37 (3,01-6,30)	4,89 (3,55-6,11)	5,63 (3,48-9,10)	4,52 (3,13-5,71)	3,92 (2,63-5,63)	4,30 (3,21-6,10)	< 0,001
L-10	0,76 (0,50-1,19)	0,87 (0,63-1,49)	0,78 (0,44-1,12)	0,66 (0,14-1,08)	0,76 (0,53-1,19)	0,79 (0,50-1,23)	< 0,001
IL-13	0,15 (0,05-0,32)	0,27 (0,13-0,52)	0,17 (0,05-0,37)	0,26 (0,10-0,41)	0,14 (0,03-0,26)	0,12 (0,06-0,27)	< 0,001
Fatores de crescimento							
FGF	3,51 (2,78-4,47)	3,78 (3,15-4,47)	3,76 (2,80-4,73)	3,07 (2,08-3,87)	3,39 (2,80-4,32)	3,69 (2,78-4,7)	< 0,001
PDGF	439,28 (184,61-827,59)	518,79 (296,67-882,61)	726,15 (287,85-1540,51)	670,21 (306,41-1102,04)	357,98 (164,63-613,35)	318,01 (132,08-662,95)	< 0,001
VEGF	7,07 (1,21-13,89)	7,61 (4,60-14,16)	8,30 (2,11-3,89)	3,42 (0,001-6,93)	7,31 (1,21-15,15)	5,82 (1,12-14,73)	< 0,001
GM-CSF	0,30 (0,19-0,44)	0,34 (0,28-0,48)	0,33 (0,13-0,56)	0,26 (0,001-0,39)	0,28 (0,18-0,40)	0,33 (0,23-0,45)	0,001
G-CSF	0,001 (0,001-1,52)	0,001 (0,001-0,84)	0,001 (0,001-1,39)	0,001 (0,001-0,95)	0,001 (0,001-1,54)	0,29 (0,001-2,15)	0,367
IL-7	1,28 (0,47-2,47)	1,43 (0,75-2,92)	1,82 (0,79-3,55)	1,46 (0,56-2,69)	1,00 (0,10-1,86)	1,45 (0,59-2,39)	0,001
IL-2	1,22 (0,80-1,91)	1,10 (0,76-1,81)	1,24 (0,85-1,89)	0,99 (0,63-1,73)	1,22 (0,80-1,88)	1,50 (0,86-2,35)	0,259

Nota: os níveis de biomarcadores foram expressos no percentil 50 (percentis 25 e 75) em pg/mL. Para estimar o valor de p foi realizado o teste Kruskall-Wallis, considerando o desenho amostral complexo da pesquisa.

As associações entre os biomarcadores categorizados em tercis e a região de residência, ajustadas pelos fatores de confusão, estão descritas na Tabela S1 (Material Suplementar; https://cadernos.ensp.fiocruz.br/static//arquivo/suppl-e00172124_2029.pdf). Em relação às quimiocinas, em comparação à Região Norte, residentes no Centro-oeste apresentaram menores chances de apresentarem níveis mais elevados de CXCL8 e CCL3, enquanto o CXCL10 apresentou menores dosagens no Sudeste e Sul. Maior chance de níveis intermediários de CCL5 (2º tercil) foi observada no Nordeste. Entre as citocinas inflamatórias, maiores chances de níveis elevados de IL-1β foram observadas nas regiões Nordeste e Sul, em comparação ao Norte. Por outro lado, menores chances de níveis mais elevados de IL-6, IL-12 e IL-17 foram observados nas regiões Centro-oeste e Sudeste; a IL-12 também apresentou menores valores no Nordeste. De maneira geral, menores chances de níveis intermediários (2º tercil) e/ou mais elevados (3º tercil) das citocinas reguladoras e fatores de crescimento foram observadas na maioria das regiões, em comparação ao Norte. Apenas a citocina IL-9 e o fator G-CSF não apresentaram diferenças significativas entre as regiões.

**Figura 1:**
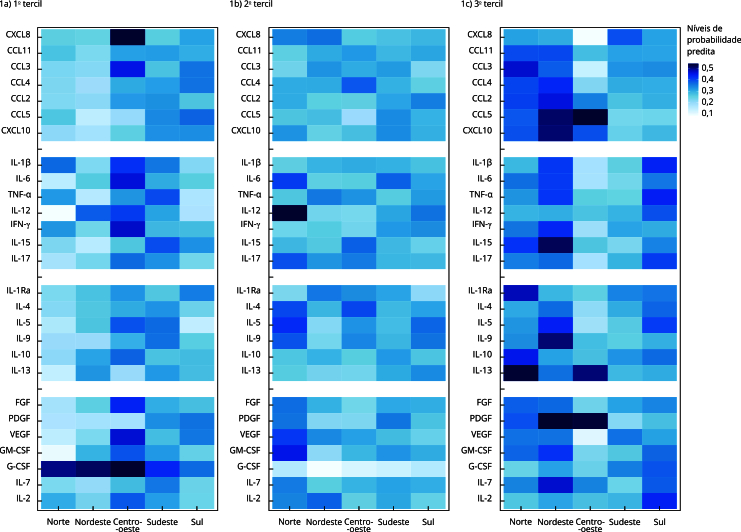
Probabilidades preditas de os biomarcadores apresentarem níveis de concentração no 1º, 2º e 3º tercis para cada região.

A partir dos modelos de regressão, foram estimadas as probabilidades preditas de os biomarcadores apresentarem níveis de concentração no 1º, 2º ou 3º tercis para cada região, sendo ajustadas pelos fatores de confusão incluídos no estudo (Figura 1). No *heatmap*, as cores mais escuras sinalizam maior a probabilidade de pertencimento a cada um dos tercis da distribuição. De modo geral, ao analisar descritivamente a probabilidade predita do 3º tercil, ou seja, a probabilidade de detecção de níveis mais elevados, observa-se que os biomarcadores imunológicos tendem a apresentar níveis mais baixos na Região Centro-oeste e mais altos nas regiões Norte e Nordeste (Figura 1).

Nota: probabilidades preditas de o biomarcador estar no 1º, 2º ou 3º tercis da distribuição, segundo as regiões geográficas brasileiras, ajustadas pelas variáveis apresentadas na Tabela 1. Os biomarcadores imunológicos estão distribuídos nas linhas e as regiões geográficas nas colunas. O gradiente da cor azul mostra diferentes níveis de probabilidade predita: quanto mais escuro maior a probabilidade e quanto mais claro menor a probabilidade predita de o biomarcador estar naquele tercil. Não houve associação estatisticamente significativa entre os biomarcadores CCL4, IL-1Ra, IL-4 ou G-GSF e as regiões geográficas.

Na análise do grupo de quimiocinas, verificou-se uma maior probabilidade predita de níveis séricos elevados (3º tercil) no Nordeste, como a CCL2, CCL5 e CXCL10. Além disso, a quimiocina CCL2, CCL5 e CXCL10 também demonstraram concentrações mais altas na Região Centro-oeste, enquanto a CXCL8 apresentou menor probabilidade de níveis elevados nessa região (Figura 1).

Em relação às citocinas inflamatórias, observou-se uma maior probabilidade predita de apresentarem níveis séricos mais baixos nas regiões Centro-oeste e Sudeste (menores probabilidades preditas no 3º tercil). Em contrapartida, as citocinas inflamatórias, em geral, demonstraram uma maior probabilidade de níveis séricos mais elevados nas regiões Nordeste e Sul, como IL-1β, IL-6 e IL-17, por exemplo (Figura 1).

Na avaliação do *heatmap*, não foi identificado um padrão claro sobre as citocinas reguladoras. No entanto, destaca-se que as citocinas IL-5 e IL-9 mostraram uma maior probabilidade de níveis elevados na Região Nordeste, enquanto IL-10 e IL-13 apresentaram essa tendência na Região Norte, com a IL-13 também possuindo probabilidade de níveis mais altos na Região Centro-oeste (Figura 1).

Assim como para as citocinas reguladoras, não foi observado um padrão claro no 3º tercil para os fatores de crescimento. No entanto, observou-se que o PDGF apresenta maior probabilidade de níveis séricos elevados nas regiões Nordeste e Centro-oeste (Figura 1). 

## Discussão

Neste trabalho avaliamos pela primeira vez o perfil geral de biomarcadores imunológicos (citocinas, quimiocinas e fatores de crescimento) e as diferenças entre as regiões geográficas brasileiras em amostra nacional representativa de adultos mais velhos. A Região Norte apresentou, em geral, maior probabilidade de apresentar marcadores inflamatórios e reguladores mais elevados. As regiões Nordeste e Sul exibiram maior probabilidade de apresentar mais grupos de marcadores inflamatórios no maior tercil. Por outro lado, a Região Centro-oeste apresentou maior probabilidade de marcadores inflamatórios estarem no menor tercil. Em síntese, nossos resultados demonstraram de forma geral que, independentemente de sua função, os níveis séricos de biomarcadores imunológicos têm maior probabilidade de estarem elevados nas regiões Norte e Nordeste do Brasil e mais baixos na Região Centro-oeste. Esses diferentes perfis refletem uma imunobiografia possivelmente influenciada pelas diferenças regionais, como as ambientais, socioeconômicas, étnicas, de exposições a patógenos, entre outras ^27^.

Entre todos os analitos, CXCL8 foi a quimiocina que apresentou maior probabilidade de níveis séricos mais baixos entre os indivíduos mais velhos residentes da Região Centro-oeste. Esse biomarcador tem um papel chave no desenvolvimento da inflamação, sendo identificado como um fator quimiotático potente e específico para neutrófilos ^36^ ^,^ ^37^ ^,^ ^38^ ^,^ ^39^. Em contrapartida, CXCL10 apresentou maior probabilidade de ter níveis elevados nessa região, bem como nas regiões Norte e Nordeste. Essa quimiocina é fortemente induzida por interferon e está associada a resposta de células T do tipo 1 (Th1), mas é um quimioatraente fraco para neutrófilos ^40^. Assim, sugere-se que há um potencial menor de recrutamento neutrofílico entre adultos mais velhos da Região Centro-oeste, o que poderia refletir em respostas infecciosas dependentes de neutrófilos menos potentes.

O envelhecimento é caracterizado por uma série de alterações fisiológicas em indivíduos mais velhos, incluindo o fenômeno *inflammaging*, marcado pelo aumento de biomarcadores inflamatórios durante a imunossenescência ^4^. As citocinas inflamatórias IL-1β e IL-17 estão fortemente associadas com o envelhecimento. O perfil dessas citocinas foi semelhante, apresentando a probabilidade de níveis mais elevados nas regiões Nordeste e Sul e mais baixos nas regiões Centro-oeste e Sudeste ^5^ ^,^ ^41^.

A citocina inflamatória IL-6 é reconhecida desde 1993 como a “citocina do envelhecimento”, e tem sido considerada um importante preditor no desenvolvimento e progressão de doenças relacionadas à idade. Uma das principais fontes celulares de IL-6 são os fibroblastos, e o FGF (fator de crescimento para fibroblastos) demonstrou um perfil de probabilidade predita semelhante ao de IL-6. Observou-se que esses biomarcadores tendem a apresentar concentração mais elevadas nas regiões Norte e Nordeste e mais baixas nas regiões Centro-oeste e Sudeste. Níveis elevados de IL-6 contribuem para um microambiente inflamado, associado a piores prognósticos em doenças crônicas, autoimunes e maior ocorrência de hospitalizações, destacando a necessidade de atenção nas regiões com níveis elevados desses biomarcadores ^38^ ^,^ ^41^ ^,^ ^42^ ^,42,^ ^43^.

A citocina IL-10 é um importante e potente biomarcador anti-inflamatório que está envolvido em diversos tipos de respostas celulares inatas e adaptativas do sistema imunológico ^44^. Observamos que existe uma maior probabilidade dessa citocina apresentar níveis elevados na Região Norte. Estudos demonstram que essa citocina pode ter efeitos relacionados ao chamado *anti-inflammaging*, que é um balanço entre os biomarcadores inflamatórios e anti-inflamatórios em idosos. O *anti-inflammaging* pode proporcionar maior longevidade, auxiliando nas respostas contra doenças crônicas inerentes ao envelhecimento, como cânceres e eventos cardiovasculares ^5^. No entanto, outros estudos demonstraram que níveis mais altos de IL-10 em idosos podem estar associados a uma resistência diminuída contra infecções, como citomegalovírus (CMV), gripes, COVID-19, helmintíases e algumas alergias ^45^ ^,^ ^46^ ^,^ ^47^. Dessa forma, seria interessante aprofundar a investigação sobre a relação entre a IL-10 e o perfil de saúde da população da Região Norte do país.

As citocinas reguladoras IL-5 e IL-9 apresentaram maior probabilidade de níveis sorológicos mais altos na Região Nordeste e mais baixos no Centro-oeste e no Sudeste; já IL-13 apresentou maior probabilidade de ter níveis elevados nas regiões Norte e Centro-oeste. Essas três citocinas possuem funções semelhantes, que estão relacionadas à produção da Imunoglobulina do tipo E (IgE), às respostas específicas contra alérgenos, ao recrutamento de eosinófilos, à produção de muco e à migração tecidual de linfócitos T com respostas do tipo 2 (Th2), além da regulação da integridade da barreira epitelial ^38^ ^,^ ^48^. Esses biomarcadores estão fortemente envolvidos em respostas contra infecções helmínticas, assim como alergias respiratórias e do trato intestinal. Portanto, essas informações poderiam ser úteis para futuras análises sobre respostas Th2, especialmente nas regiões Norte, Nordeste e Centro-oeste, onde essas citocinas apresentaram níveis elevados.

As quimiocinas CCL2 e CCL5 apresentaram maior probabilidade de níveis elevados nas regiões Nordeste e Centro-oeste, enquanto CCL3 apresentou maior probabilidade de níveis elevados na Região Norte e menores no Centro-oeste. Em geral, as quimiocinas da família CC desempenham um papel crucial no processo inflamatório, participando de ações quimiotáticas que envolvem monócitos, células Natural Killers (NK) e linfócitos T. A quimiocina CCL2 é importante na diferenciação de células Th2, enquanto CCL3 e CCL5 de células Th1 ^40^ ^,^ ^49^. Estudos realizados no Brasil demonstraram associações positivas entre níveis elevados dessas duas quimiocinas e parasitoses, como doença de Chagas e hanseníase ^12^ ^,^ ^13^ ^,^ ^50^. Essas diferenças entre as regiões podem refletir em um perfil imunológico da população brasileira que ainda convive com a presença de uma série de doenças negligenciadas, como esquistossomose, leishmaniose e febre amarela ^11^. 

O PDGF (fator de crescimento derivado de plaquetas) está relacionado a uma série de processos biológicos, como angiogênese, cicatrização de feridas e desenvolvimento de doenças crônicas. Esse fator desempenha um papel importante na calcificação vascular, uma vez que seus níveis elevados estão associados à reconstrução adversa dos vasos sanguíneos ^48^ ^,^ ^51^. No presente estudo, o PDGF foi o único fator de crescimento que apresentou maior probabilidade de ter níveis elevados na Região Centro-oeste, enquanto os outros fatores de crescimento analisados mostraram tendência a níveis séricos mais baixos nessa mesma região. Além disso, o PDGF também mostrou maior probabilidade de apresentar níveis séricos elevados nas regiões Norte e Nordeste.

De modo geral, pouco se sabe sobre a relação entre fatores de crescimento, como GM-CSF (fator de crescimento para granulócitos), IL-7 (para linfócitos B), IL-2 (para linfócitos T) e VEGF (fator de crescimento endotelial vascular), e o processo de envelhecimento no Brasil. Diante dos resultados observados, principalmente na Região Centro-oeste, acreditamos que novos estudos são necessários para avaliar possíveis associações entre esses fatores de crescimento e o envelhecimento.

Estudos no Brasil têm demonstrado variações regionais significativas em relação à ancestralidade e às condições socioeconômicas. A população brasileira apresenta um perfil de ancestralidade mista, incluindo ascendências europeia, africana e nativo-americana. A iniciativa *Epidemiologia Genômica de Coortes Brasileiras* (EPIGEN-Brasil), que analisou três coortes em cidades de diferentes portes (Bambuí, Minas Gerais; Pelotas, Rio Grande do Sul; e Salvador, Bahia), localizadas nas três regiões mais populosas do país, revelou que Pelotas (Região Sul) possui maior ancestralidade europeia, enquanto Salvador (Região Nordeste) apresenta maior ancestralidade africana ^52^. Adicionalmente, Ruiz-Linares et al. ^53^ identificaram que a maior predominância de ancestralidade nativo-americana está concentrada na Região Amazônica, no Norte do Brasil ^16^ ^,^ ^52^ ^,^ ^53^. Além das questões de ancestralidade, as desigualdades socioeconômicas são uma realidade marcante no Brasil, impactando significativamente os serviços de saúde, especialmente nas regiões Norte e Nordeste, onde o sistema de saúde é mais precário. Essas desigualdades se manifestam nas barreiras ao acesso aos cuidados médicos, na maior prevalência de doenças crônicas e nas discrepâncias na expectativa de vida saudável, refletindo as disparidades socioeconômicas regionais ^20^ ^,^ ^21^ ^,^ ^54^ ^,^ ^55^. 

Não está claro se os efeitos sobre ancestralidade e desigualdades estão diretamente correlacionados com as diferenças regionais brasileiras e a distribuição de citocinas, quimiocinas e fatores de crescimento. No entanto, esses fatores podem explicar parcialmente essas diferenças, uma vez que as análises consideraram ajustes por fatores de confusão, que incluem aspectos socioeconômicos, estilo de vida e condições de saúde dos adultos mais velhos brasileiros.

Entre as limitações deste estudo, podemos citar o fato de que os ensaios foram realizados in vitro, o que impede a observação direta das interações entre os efeitos biológicos e fisiológicos e os biomarcadores imunológicos avaliados. Além disso, não temos informações sobre infecções virais, bacterianas e parasitárias. No entanto, este estudo é pioneiro na avaliação das diferenças entre as cinco regiões brasileiras em termos de distribuição de biomarcadores imunológicos séricos. Investigações mais aprofundadas sobre as possíveis associações entre esse perfil imunológico e outros fatores sociodemográficos poderão contribuir para um entendimento mais robusto das diferenças reportadas nesse estudo. 

Em síntese, este trabalho demonstrou que existem diferenças importantes no perfil dos biomarcadores avaliados entre adultos mais velhos e as regiões brasileiras. Observou-se que os participantes do ELSI-Brasil têm maior probabilidade de apresentar níveis séricos mais altos nas regiões Norte e Nordeste, enquanto a Região Centro-oeste tende a apresentar níveis mais baixos. As razões dessas diferenças precisam ser investigadas em estudos posteriores que avaliem as associações entre os biomarcadores destacados e desfechos de saúde específicos.

## Data Availability

Os dados de pesquisa estão disponíveis mediante solicitação à autora de correspondência.

## Material Suplementar

Material Suplementar
