# Supplementary material for: Evaluation of the immunological profile of older adults according to Brazilian regions: results of ELSI-Brazil
Source: Cad Saude Publica. 2026 May 29;42:e00172124. [Article in Portuguese] doi: 10.1590/0102-311XPT172124 (PMC13224955; doi:10.1590/0102-311XPT172124)
Supplement: Material Suplementar [file 1678-4464-csp-42-PT172124-s.pdf]

# Material Suplementar

**Tabela S1** *Odds ratio* (OR) e intervalos de 95% de confiança (IC95%) ajustados das associações entre cada biomarcador em tercis (desfecho) e as regiões geográficas (exposição). *Estudo Longitudinal da Saúde dos Idosos Brasileiros* (ELSI-Brasil), 2015-2016.

| Biomarcadores | Nordeste | Centro-oeste | Sudeste | Sul |
|---|---|---|---|---|
| **Quimiocinas** | | | | |
| CXCL8 | | | | |
| 2º tercil | 1,087<br>(0,288-4,106) | 0,345<br>(0,100-1,183) | 0,779<br>(0,234-2,595) | 0,673<br>(0,174-2,606) |
| 3º tercil | 0,988<br>(0,369-2,642) | 0,118 *<br>(0,024-0,576) | 1,245<br>(0,462-3,355) | 0,809<br>(0,165-3,972) |
| CCL11 | | | | |
| 2º tercil | 1,462<br>(0,954-2,240) | 1,106<br>(0,711-1,721) | 0,976<br>(0,614-1,551) | 1,105<br>(0,575-2,122) |
| 3º tercil | 1,227<br>(0,662-2,275) | 0,604<br>(0,329-1,109) | 0,666<br>(0,373-1,189) | 0,705<br>(0,232-2,136) |
| CCL3 | | | | |
| 2º tercil | 1,443<br>(0,778-2,677) | 0,613<br>(0,348-1,080) | 1,114<br>(0,645-1,923) | 0,586<br>(0,283-1,217) |
| 3º tercil | 0,830<br>(0,361-1,910) | 0,131 *<br>(0,058-0,297) | 0,554<br>(0,251-1,224) | 0,428<br>(0,096-1,901) |
| CCL4 | | | | |
| 2º tercil | 1,160<br>(0,458-2,935) | 0,950<br>(0,394-2,293) | 0,704<br>(0,306-1,624) | 0,521<br>(0,185-1,468) |
| 3º tercil | 1,247<br>(0,458-3,397) | 0,394<br>(0,145-1,072) | 0,529<br>(0,231-1,213) | 0,457<br>(0,128-1,638) |
| CCL2 | | | | |
| 2º tercil | 0,872<br>(0,495-1,534) | 0,576<br>(0,319-1,040) | 0,685<br>(0,439-1,070) | 0,946<br>(0,546-1,639) |
| 3º tercil | 1,172<br>(0,624-2,203) | 0,594<br>(0,325-1,083) | 0,445 *<br>(0,243-0,815) | 0,594<br>(0,166-2,122) |
| CCL5 | | | | |
| 2º tercil | 1,824 *<br>(1,238-2,688) | 0,968<br>(0,564-1,662) | 0,990<br>(0,637-1,539) | 0,777<br>(0,444-1,362) |
| 3º tercil | 2,367<br>(0,646-8,670) | 1,893<br>(0,520-6,897) | 0,475<br>(0,141-1,607) | 0,434<br>(0,096-1,962) |
| CXCL10 | | | | |
| 2º tercil | 0,889<br>(0,634-1,247) | 0,686<br>(0,468-1,005) | 0,626 *<br>(0,431-0,910) | 0,549 *<br>(0,339-0,889) |
| 3º tercil | 1,564<br>(0,587-4,165) | 0,816<br>(0,376-1,772) | 0,367 *<br>(0,157-0,859) | 0,428<br>(0,140-1,310) |
| **Citocinas inflamatórias** | | | | |
| IL-1β | | | | |
| 2º tercil | 1,982<br>(0,835-4,704) | 1,052<br>(0,485-2,284) | 1,242<br>(0,542-2,847) | 1,897<br>(0,654-5,502) |
| 3º tercil | 2,507 *<br>(1,310-4,798) | 0,542<br>(0,197-1,494) | 0,927<br>(0,413-2,079) | 2,691 *<br>(1,106-6,545) |
| IL-6 | | | | |
| 2º tercil | 0,355 *<br>(0,185-0,679) | 0,200 *<br>(0,103-0,390) | 0,461 *<br>(0,230-0,922) | 0,442<br>(0,148-1,319) |
| 3º tercil | 0,657<br>(0,284-1,523) | 0,153 *<br>(0,076-0,307) | 0,313*<br>(0,140-0,699) | 0,569<br>(0,148-2,186) |
| TNF-α | | | | |
| 2º tercil | 2,800<br>(0,897-8,734) | 1,225<br>(0,426-3,519) | 0,775<br>(0,266-2,259) | 2,356<br>(0,638-8,700) |
| 3º tercil | 2,723<br>(0,583-12,726) | 0,785<br>(0,186-3,304) | 0,600<br>(0,133; 2,696) | 2,718<br>(0,536-13,776) |
| IL-12 | | | | |
| 2º tercil | 0,100*<br>(0,031-0,317) | 0,091 *<br>(0,033-0,250) | 0,162 *<br>(0,056-0,467) | 0,338<br>(0,109-1,054) |
| 3º tercil | 0,243 *<br>(0,099-0,597) | 0,204 *<br>(0,098-0,425) | 0,290 *<br>(0,128-0,661) | 0,687<br>(0,275-1,714) |
| IFN-γ | | | | |

| | | | | |
|---|---|---|---|---|
| 2º tercil | 1,525 | 0,666 | 1,527 | 1,611 |
| | (0,466-4,984) | (0,237-1,873) | (0,621-3,752) | (0,422-6,147) |
| 3º tercil | 1,667 | 0,359 | 0,971 | 0,961 |
| | (0,444-6,258) | (0,081-1,590) | (0,236-3,991) | (0,153-6,034) |
| IL-15 | | | | |
| 2º tercil | 1,319 | 1,092 | 0,549 | 0,572 |
| | (0,526-3,307) | (0,338-3,531) | (0,234-1,287) | (0,220-1,488) |
| 3º tercil | 1,765 | 0,529 | 0,295 | 0,545 |
| | (0,422-7,388) | (0,124-2,251) | (0,070-1,250) | (0,094-3,142) |
| IL-17 | | | | |
| 2º tercil | 0,661 | 0,443 * | 0,412 * | 0,578 |
| | (0,372-1,176) | (0,259-0,756) | (0,245-0,691) | (0,298-1,120) |
| 3º tercil | 0,846 | 0,243 * | 0,461 | 0,939 |
| | (0,345-2,077) | (0,071-0,832) | (0,184-1,155) | (0,272-3,240) |
| **Citocinas reguladoras** | | | | |
| IL-1Ra | | | | |
| 2º tercil | 1,297 | 1,016 | 1,157 | 0,590 |
| | (0,751-2,239) | (0,583-1,770) | (0,686-1,950) | (0,277-1,258) |
| 3º tercil | 0,490 | 0,354 * | 0,595 | 0,467 |
| | (0,230-1,046) | (0,176-0,710) | (0,287-1,235) | (0,109-1,999) |
| IL-4 | | | | |
| 2º tercil | 0,591 | 0,716 | 0,478 * | 0,705 |
| | (0,290-1,205) | (0,358-1,432) | (0,239-0,957) | (0,232-2,140) |
| 3º tercil | 0,984 | 0,475 | 0,650 | 1,111 |
| | (0,301-3,220) | (0,101-2,237) | (0,225-1,877) | (0,226-5,469) |
| IL-5 | | | | |
| 2º tercil | 0,317 * | 0,328 * | 0,299 * | 1,017 |
| | (0,162-0,619) | (0,178-0,605) | (0,141-0,630) | (0,397-2,606) |
| 3º tercil | 0,875 | 0,248 * | 0,367 | 1,49 |
| | (0,281-2,726) | (0,078-0,786) | (0,105-1,280) | (0,366-6,070) |
| IL-9 | | | | |
| 2º tercil | 0,598 | 0,600 | 0,413 | 0,730 |
| | (0,192-1,856) | (0,200-1,798) | (0,133-1,285) | (0,240-2,216) |
| 3º tercil | 1,553 | 0,572 | 0,425 | 0,694 |
| | (0,632-3,818) | (0,259-1,264) | (0,177-1,019) | (0,226-2,134) |
| L-10 | | | | |
| 2º tercil | 0,748 | 0,545 | 0,925 | 0,756 |
| | (0,185-3,022) | (0,162-1,833) | (0,298-2,872) | (0,222-2,574) |
| 3º tercil | 0,468 | 0,341 * | 0,552 | 0,605 |
| | (0,201-1,090) | (0,137-0,849) | (0,214-1,424) | (0,210-1,746) |
| IL-13 | | | | |
| 2º tercil | 0,398 * | 0,648 | 0,529 * | 0,672 |
| | (0,228-0,692) | (0,357-1,176) | (0,299-0,936) | (0,222-2,034) |
| 3º tercil | 0,297 * | 0,674 | 0,234 * | 0,285 |
| | (0,133-0,666) | (0,301-1,509) | (0,105-0,519) | (0,076-1,075) |
| **Fatores de crescimento** | | | | |
| FGF | | | | |
| 2º tercil | 0,592 | 0,269 * | 0,511 * | 0,542 |
| | (0,292-1,200) | (0,130-0,559) | (0,262-0,995) | (0,197-1,493) |
| 3º tercil | 0,713 | 0,252 * | 0,502 | 0,595 |
| | (0,333-1,526) | (0,104-0,608) | (0,242-1,040) | (0,175-2,021) |
| PDGF | | | | |
| 2º tercil | 0,579 * | 0,590 | 0,525 * | 0,363 * |
| | (0,335-1,000) | (0,324-1,074) | (0,321-0,858) | (0,214-0,616) |
| 3º tercil | 1,355 | 1,332 | 0,287 * | 0,336 |
| | (0,465-3,951) | (0,474-3,747) | (0,128-0,646) | (0,111-1,012) |
| VEGF | | | | |
| 2º tercil | 0,549 | 0,241 | 0,365 | 0,250 |
| | (0,085-3,525) | (0,039-1,474) | (0,062-2,145) | (0,036-1,731) |
| 3º tercil | 0,675 | 0,098 * | 0,545 | 0,371 |
| | (0,286-1,590) | (0,035-0,274) | (0,250-1,187) | (0,094-1,467) |
| GM-CSF | | | | |
| 2º tercil | 0,156 * | 0,160 * | 0,220 * | 0,267 * |
| | (0,043-0,562) | (0,072-0,356) | (0,099-0,485) | (0,118-0,604) |
| 3º tercil | 0,385 * | 0,153 * | 0,219 * | 0,411 |
| | (0,168-0,880) | (0,061-0,385) | (0,088-0,543) | (0,102-1,659) |
| G-CSF | | | | |
| 2º tercil | 0,517 | 0,694 | 0,955 | 1,367 |
| | (0,159-1,686) | (0,210-2,293) | (0,315-2,893) | (0,305-6,130) |
| 3º tercil | 1,249 | 0,916 | 1,663 | 2,2 |

| | | | | |
|---|---|---|---|---|
| | (0,380-4,100) | (0,287-2,922) | (0,571-4,845) | (0,533-9,077) |
| IL-7 | | | | |
| 2º tercil | 0,795 | 0,660 | 0,561 * | 0,787 |
| | (0,374-1,687) | (0,406-1,073) | (0,319-0,986) | (0,335-1,850) |
| 3º tercil | 1,496 | 0,798 | 0,441 * | 1,041 |
| | (0,832-2,689) | (0,430-1,481) | (0,213-0,915) | (0,406-2,671) |
| IL-2 | | | | |
| 2º tercil | 1,427 | 0,569 * | 0,849 | 0,913 |
| | (0,656-3,100) | (0,337-0,961) | (0,455-1,584) | (0,395-2,111) |
| 3º tercil | 1,506 | 0,799 | 1,032 | 2,108 |
| | (0,820-2,763) | (0,377-1,694) | (0,492-2,165) | (0,737-6,032) |

Nota: OR e IC95% em tercis, ajustados por idade, sexo, nível educacional, área de residência, consumo de álcool, consumo de tabaco, prática de atividade física e os diagnósticos médicos autorreferidos, hipertensão, diabetes, asma, artrite e câncer. Categorias de referência: Região Norte e 1º tercil.

* Valor de $p < 0,05$.
